# Alternating Magnetic Field-Promoted Nanoparticle Mixing: The On-Chip Immunocapture of Serum Neuronal Exosomes for Parkinson’s Disease Diagnostics

**DOI:** 10.1021/acs.analchem.3c00357

**Published:** 2023-05-11

**Authors:** Mohamed Sharafeldin, Shijun Yan, Cheng Jiang, George K. Tofaris, Jason J. Davis

**Affiliations:** † Department of Chemistry, University of Oxford, South Parks Road, Oxford OX1 3QZ, U.K.; ‡ Department of Chemistry, University of Otago, Dunedin 9054, New Zealand; § Nuffield Department of Clinical Neurosciences, John Radcliffe Hospital, University of Oxford, Oxford OX3 9DU, U.K.; ∥ Kavli Institute for Nanoscience Discovery, Dorothy Crowfoot Hodgkin Building, University of Oxford, Oxford OX1 3QU, U.K.

## Abstract

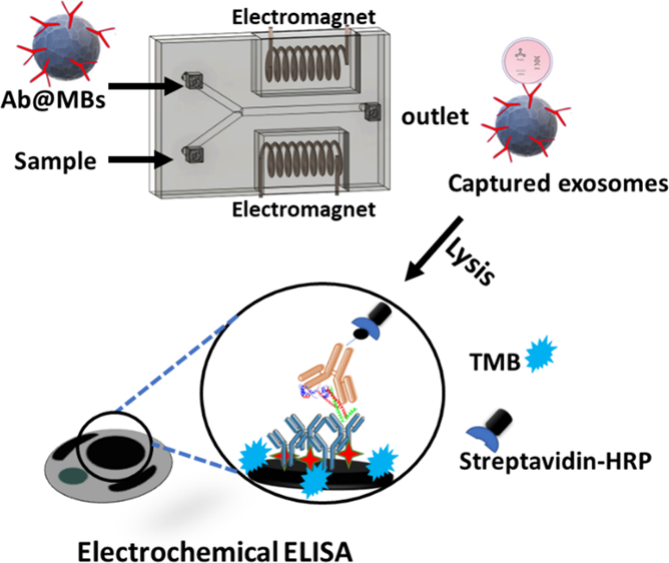

The analysis of cargo proteins in exosome subpopulations has considerable value in diagnostics but a translatable impact has been limited by lengthy or complex exosome extraction protocols. We describe herein a scalable, fast, and low-cost exosome extraction using an alternating (AC) magnetic field to support the dynamic mixing of antibody-coated magnetic beads (MBs) with serum samples within 3D-printed microfluidic chips. Zwitterionic polymer-coated MBs are, specifically, magnetically agitated and support ultraclean exosome capture efficiencies >70% from <50 μL of neat serum in 30 min. Applied herein to the immunocapture of neuronal exosomes using anti-L1CAM antibodies, prior to the array-based assaying of α-synuclein (α-syn) content by a standard duplex electrochemical sandwich ELISA, sub pg/mL detection was possible with an excellent coefficient of variation and a sample-to-answer time of ∼75 min. The high performance and semiautomation of this approach hold promise in underpinning low-cost Parkinson’s disease diagnostics and is of value in exosomal biomarker analyses more generally.

## Introduction

Exosomes, a subtype of extracellular vesicles 30–150 nm in size, are secreted by most cell types and found in all biofluids. Because they are generated within cells, they are considered to represent a snapshot of the cellular state and have thus generated extensive interest as potential biomarkers.^1,2^ They carry an array of genetic material, lipids, and proteins that can report on the origin and status of the parent cells.^3−6^ Those released from neuronal cells (neuronal exosomes) can be detected in the circulation,^7^ where they can potentially be used as proxy biomarkers for the pathophysiological state of neurons.^8−11^ We have previously shown that total alpha-synuclein (α-syn) content of L1CAM-positive exosomes, isolated by immunocapture, can differentiate patients with Parkinson’s disease (PD) or at-risk individuals such as those with REM sleep behavior disorder from controls and patients with atypical Parkinson’s.^12,13^

PD is characterized pathologically by the accumulation and aggregation of α-syn (synucleinopathy) in intraneuronal inclusions termed Lewy bodies.^14−16^ While the incidence of PD is increasing worldwide, its diagnosis is typically made when patients present with clinical symptoms and already have extensive pathology. Biomarkers such as exosomal α-syn that can offer early diagnosis, before advanced neurodegeneration, will ultimately enable a much earlier therapeutic intervention and the application of disease modifying agents to slow progression and improve patients’ quality of life.^17^ Achieving a sensitive exosomal α-syn assay requires a robust exosome isolation method that avoids the inherent variability arising from manual sample handling during extraction and, ideally, is both highly selective and operates with very small sample volumes.^18^

Popular methods for exosome isolation are based on differential centrifugation, size exclusion, and polymer-based precipitation. These are of low specificity (enriching also cells and cell debris), laborious, and most typically require several hundred microliters of patient blood.^19,20^ The immune-affinity isolation of exosome subpopulations (based on specific surface marker expression such as L1CAM) can be effective when applied to solid supports within chromatographic or microfluidic isolation^21−23^ but these approaches can be associated with low capture yield and/or the need to generate high surface area nanostructured supports (solid pillars or porous).^24−27^ The solution phase application of dispersed antibody-coated magnetic beads (MBs) has accordingly been an increasingly popular and accessible means of exosome isolation but typically requires several (4–16) hours of incubation, sometimes with additional continuous stirring, in order to maximize immunocapture.^28^ There have been a number of attempts to improve MB mixing dynamics and associated capture kinetics (potentially enabling ∼1 h isolation from ∼100 μL of samples),^29^ for example, through the use of micromixers^30^ or nanostructured microfluidic channels,^31^ but these required both complex engineering (nanometer-sized channels or nanoarchitecture surfaces) and a secondary (offline) magnetic separation step.^32,33^ Standard MB-based exosome extraction typically utilizes well-established robust antibody coupling at chemically simple interfaces and although this can support high (70–90%) capture efficiencies, from serum or cerebrospinal fluid, it is usually associated with extensive manual sample handling and washing.^34^ Prior semiautomated on-chip exosome isolation methods have been proposed as alternatives to manual MB extraction reducing labour, time, and risk of cross-contamination.^35^ Such approaches, however, have been associated with a relatively low capture yield (<70%) that can only be practically applied to real sample analysis where exosomes are expressed at high levels.^36^ The vast majority of such approaches also employ MBs with a simple surface chemistry that will be unable to prevent the acquisition of nonspecifically adsorbed proteins, nucleic acids, or other impurities, with a potentially significant impact on downstream analyses.^37,38^ More sophisticated MB coatings are required if clean extraction is to be achieved from real samples.^39^ We previously showed that zwitterionic polymer-coated MBs support the specific recruitment of neuronal exosomes with a negligible background; this is especially relevant if one seeks to assay specific marker subpopulations. α-Syn, for example, is abundant in many cell types and in a free form in the circulation, thereby downstream analysis of exosomal α-Syn requires highly specific target exosome capture (without any contribution from free α-Syn).^40^

**Scheme 1:**
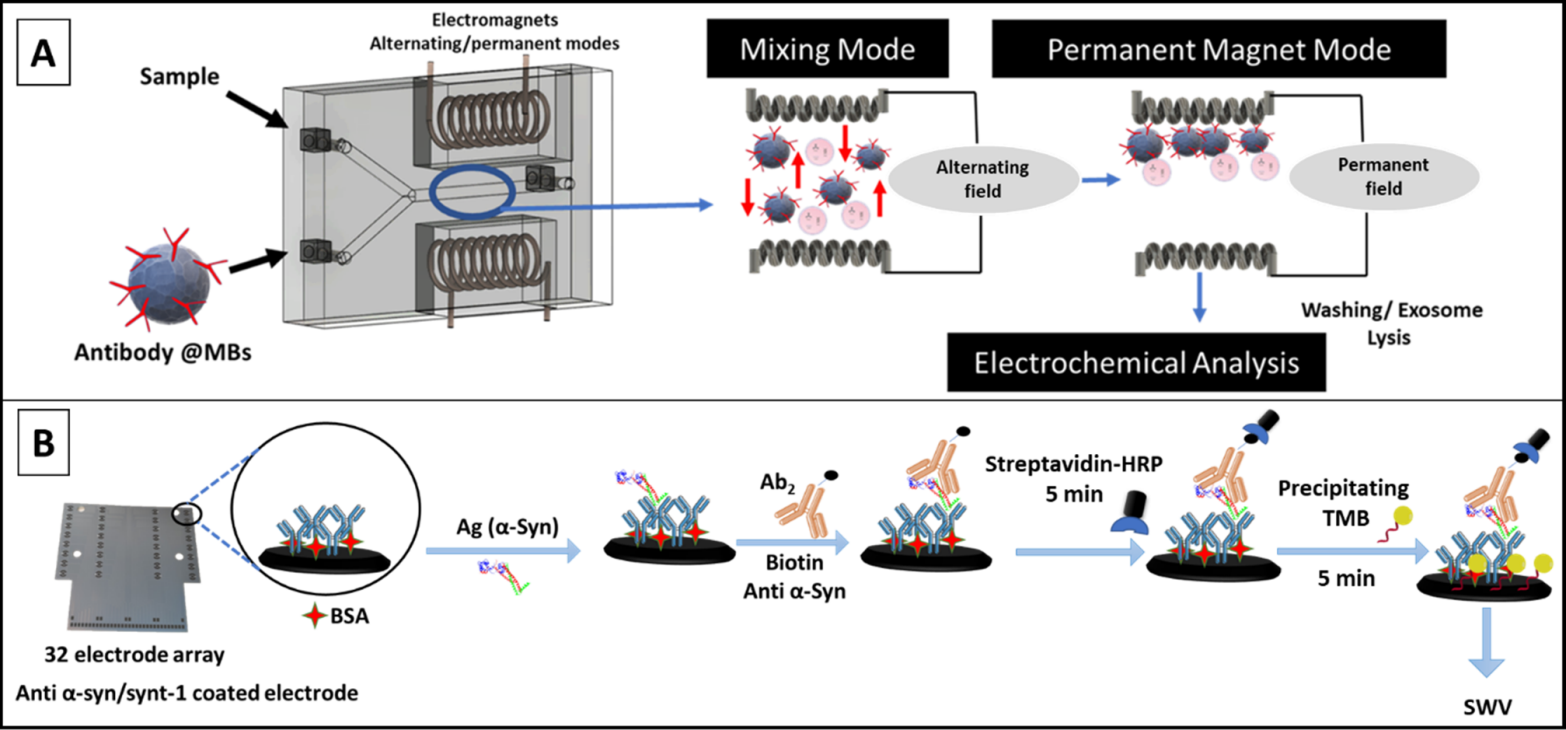
(A) Schematic Depiction of the 3D-Printed Microfluidic Chips (40 mm (*W*) × 15 mm (*L*) × 8 mm (*H*)) Housing a Y-Shaped Mixing Chamber (1.5 mm Internal Diameter) with Two Inlets, For Sample and MBs, Lying Central between Two Electromagnets (see Figure S1, SI, for More Details) and (B) Representation of the Standard Sandwich Electrochemical ELISA for α-Syn and Synt-1 on Screen-Printed 32-Electrode Arrays Electromagnet actuation was programmed (using an Arduino UNO microprocessor) to enable switching between AC and permanent modes for optimized mixing, extraction, washing, and lysis. The lysate contents were then assayed electrochemically for α-syn and synt-1. Captured target antigens were allowed to react with secondary biotinylated antibodies prior to labelling with HRP. The subsequent marker concentration-dependent precipitation of TMB on underlying electrodes is assessed by SWV.

Herein, we have sought to control the collective motion of magnetic immunoparticles through the use of alternating (AC) magnetic fields, generated by off-the-shelf electromagnets, spanning simple-printed microfluidic channels; we observe that this promotes capture efficiencies within a few tens of minutes, which are typically (otherwise) only achieved across many hours.^41^ Magnetic field switching also promotes a controlled in situ exosome washing and lysing (Scheme 1A) prior to a dual marker α-syn and syntenin-1 (synt-1) electrochemical quantification at disposable 32-multielectrode arrays (Scheme 1B). The entire methodology was validated through the analysis of 72 patient samples, with a resolved strong statistical correlation between α-syn expression in neuronal (L1CAM+) exosome and PD status. Resolved marker levels were further validated with reference to a commercial electrochemiluminescence assay (MSD) from L1CAM+ exosomes isolated manually following overnight incubation with poly(carboxybetaine methacrylate) (pCMBA)-coated immunobeads.

## Experimental Section

A detailed description of all materials and method is present in the Supporting Information (SI).

### Magnetic Nanoparticle Synthesis

Seven hundred nanometer silica-coated superparamagnetic beads (Cytiva) at 10 mg/mL in 5 mL ethanol/water (1:1 v/v) were allowed to react with 100 μL (3-aminopropyl)triethoxysilane (APTES) in presence of 100 μL of tetraethyl orthosilicate (TEOS) overnight to form APTES-modified MBs. The 740 nm APTES-MBs were extracted from the reaction solution using a magnet, washed three times with water and three times with ethanol, and then dried under vacuum; 10 mg/mL APTES-MBs (in water) were then functionalized with epichlorohydrin (100 μL) in the presence of 100 μL of tetraethylenepentamine (in an ice bath for 1 h), washed, and then incubated with the polymerization chain transfer agent (CTA) (bis(carboxymethyl) trithiocarbonate (BCTTC)) (38 mg in ethanol/water (1:1 v/v)). The particles were magnetically separated, washed with ethanol/water 50% v/v, resuspended in ethanol/water (1:1 v/v), and incubated overnight with a mixture of BCTTC (3.7 mg), 4,4′-azobis(4-cyanovaleric acid) (ACVA) (10 mg), and carboxybetaine methacrylate (CBMA) (38 mg). After incubation, particles were washed three times with water and three times with ethanol, dried under vacuum, and stored at 4 °C. Particles were resuspended in water at 2 mg/mL, and free carboxylates were activated using EDC/NHSS for 30 min, washed, and incubated with antibodies for 3 h at RT. The polymer film thickness (approximately 20 nm) and low charge (≈−5 mV) were as expected. The antibody coverage was ≈4.77 μg/mg particles representing 81% of the theoretical monolayer surface coverage. These particles showed extremely low susceptibility to nonspecific adsorption of proteins thereafter as indicated by the low background of electrochemically quantified adsorbed proteins.

### Exosome Isolation

A dual syringe pump was used to control the sample and MB injection, independently. Fifty microliters of MBs in PBS at 2 mg/mL (equiv to 100 μg MBs) were first introduced into a microfluidic chip at 20 μL/min. The magnetic field was activated in permanent mode by applying 12 V potential to the left-hand-side magnet in order to hold MBs inside a mixing chamber. Fifty microliters of the sample was then pumped into a mixing chamber at 20 μL/min under a permanent magnetic field. Once the sample filled the mixing chamber (90 s), the flow was stopped, and the magnetic field switched to alternating mode at an optimized frequency using a programmable Arduino chip with an adjustable energy supply. After 30 min of mixing, the magnetic field was switched into permanent mode and MBs were washed by flowing 200 μL of PBS-T20 at 50 μL/min (4 min). After washing, 50 μL of lysis buffer was pumped into a mixing chamber at 50 μL/min where it was incubated with MBs (carrying captured exosomes) under AC mode to allow exosome lysis. Lysates (50 μL) were then transferred into a collection tube and diluted 4× using PBS for electrochemical analysis. For exosome extraction without lysis, the same abovementioned extraction procedures were followed and the MBs with captured exosomes were transferred into a collection tube without lysis. Exosome extraction for MSD analysis was performed using the same MBs, incubated overnight at 4 °C with 250 μL of serum samples in low-binding tubes (Eppendorf) under continuous stirring. MBs were collected, washed 3× with PBS-T20 using a permanent magnet, and then incubated for 15 min with the lysis buffer before α-syn and synt-1 quantitation using the standard MSD protocol (Materials and Methods, SI).

### Electrochemical Assay

The electrochemical assay was developed on a multielectrode (32 electrodes) array for analysis of both proteins in up to 10 samples simultaneously using a standard sandwich ELISA protocol. Anti-α-syn (from Human alpha-Synuclein DuoSet ELISA (Cat # DY1338-05), R&D Systems, USA) or anti-synt-1 (from Abcam, Cat # EPR8102, Invitrogen, Cat # PA5-28813) primary antibodies (capture Ab) were coated on electrodes at optimized concentrations. Arrays (32 electrodes) were assigned to analyze either α-syn or synt-1, where each array was used to analyze 10 samples (in duplicates) while running six standard concentrations (for electrochemical signal correction). Each electrode was incubated with 50 μL sample for 15 min, washed twice with 100 μL PBS-T20, incubated with biotinylated (anti-α-syn from Human alpha-Synuclein DuoSet ELISA (Cat# DY1338-05), R&D Systems, USA, and anti-synt-1 from Novus Biologicals, Cat # K10P3D5) secondary antibodies (detection Ab) for 10 min, washed twice with 100 μL of PBS-T20, and then incubated for 10 min with 50 μL of streptavidin-HRP. Finally, electrodes were washed and incubated for 2 min with the precipitating form of tetramethylbenzidine (TMB). After washing with water, the electrochemical signal was recorded using square wave voltammetry (SWV) (0.0–0.5 V vs Ag/AgCl) with 2 mV potential steps, 20 mV amplitudes, and 25 Hz frequency. The calibration curves were established by correlating the concentration of standard recombinant proteins against SWV peak heights. For patient samples, the same procedures were followed while using eight wells/plate for electrochemical signal correction (as calibrators). α-Syn and synt-1 concentrations in each sample were then estimated using the calibration data obtained using standard recombinant proteins (corrected against measured standards on each array). While using 32-electrode arrays enabled high throughput analyses, it prohibited a single step integration of electrochemical assaying and microfluidic exosome isolation.

## Results and Discussion

### Magnetic Bead Synthesis and Characterization

pCBMA-coated MBs were synthesized using a previously reported methodology^40^ with a slight modification. The polymeric coat was specifically formed on 700 nm silica-coated (magnetic core/silica shell) MBs premodified with a 20 (±11) nm combined APTES/TEOS layer (Figure S2, SI). The so generated amine-rich periphery facilitates the covalent tethering of the BCTTC CTA. The particles were then mixed with the CBMA monomer, CTA, and initiator (ACVA) to form a 15 (±7) nm zwitterionic pCBMA coat (Figure S2, SI; see Materials and Methods, SI for the detailed synthesis protocol). The natively anionic particle zeta potentials (−17 ± 3 mV) increased to +22 (±4 mV) after silane modification and finally to −4 (±1 mV) after polymerization (Figure S2B, SI). The subsequent antibody loading was estimated at 4.8 μg/mg MBs (∼7.0 × 10^4^ Ab/particle) using a bicinchoninic acid (BCA) total protein assay (see Materials and Methods, SI) representing >80% of the theoretical monolayer surface coverage. The so-generated particles showed undetectable protein adsorption (below the BCA assay limit of detection (<0.2 μg/mL)) after incubation with excess (10 mg/mL) human serum albumin for 1 h (see Materials and Methods). The nonfouling nature of the MBs was also further examined by both downstream electrochemical analysis (see Figure 2E,F for more details) and amplified fluorescence microscopy assays (Figures S2 and S3, SI; see Materials and Methods; Immunofluorescence Assay). A western blot (WB) analysis of bare pCBMA MBs also showed no detectable recruitment of the exosome-specific proteins L1CAM and CD81 after overnight incubation in lysate (Figure S2, SI), confirming that exosome recruitment was immune-specific.

### Microfluidic Exosome Isolation

We designed, and 3D-printed a microfluidic Y-shaped mixer equipped with a double inlet for sample and immunogenic MB injection leading to a cylindrical mixing chamber of 1.5 mm ID and 50 μL volume (Scheme 1A and Figure S1, SI). The 50 μL mixing chamber was coated with 1% bovine serum albumin (BSA) in PBS before use to reduce the susceptibility of the printed surfaces to nonspecific serum protein adsorption then placed in-between two electrically activated bidirectional magnets undergoing alternate activation cycles at a rate that was tuneable between 2 and 0.25 Hz using a power source of specific potential (8–16 V). MBs coated with either anti-CD9 or anti-L1CAM antibodies were used to extract CD9+ exosomes (representing some 75–85% of the generic serum exosome population)^42^ or L1CAM+ exosomes (8–13% of the total exosome population).^40,42^

**Figure 1:**
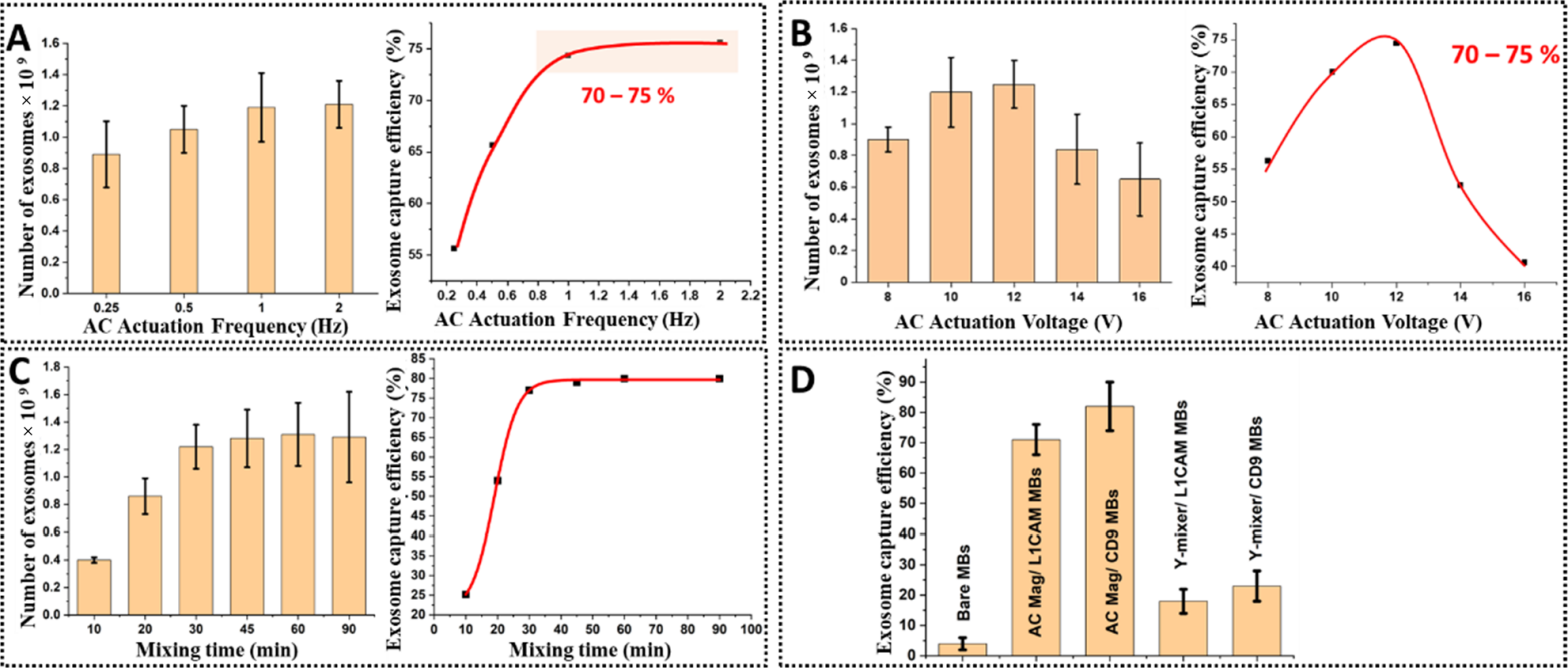
(A) Effects of AC actuation frequency and (B) actuation potential on exosome isolation efficiencies from diluted human serum. The highest efficiencies were realized at 1 Hz and 12 V actuation. (C) Effect of mixing time inside a microfluidic mixer (AC magnetic mixing) on exosome isolation efficiency. At 30 min mixing, this is optimized and not observed to improve significantly at longer (up to 90 min) incubation (70–78%). (D) NTA resolved exosome capture efficiencies with bare MBs, anti-L1CAM, and anti-CD9-coated MBs under AC magnetic and passive mixing. Captured exosomes were quantified after elution from MBs using glycine-HCl buffer (pH 2.5); the L1CAM+ and CD9+ subpopulations were estimated at 11 and 75% of the total exosome count, respectively (consistent with previous reports).^42^ Error bars represent one SD across nine NTA measurements/parameter.

Both activation potential and frequency determine the fluid dynamics and mixing and are independently controlled; these were optimized at 1 Hz with 12 V actuation as assessed by nanoparticle tracking analysis (NTA) (Figure 1A,B). Mixing time variance was also evaluated with a 30 min incubation supporting an approximate 70–75% extraction efficiency (Figure 1C); shorter incubation times resulted in reduced capture efficiency, while longer incubation did not result in a significant improvement. To isolate captured exosomes from other serum components, the magnetic field was switched to permanent mode trapping the MBs (with their cargo) under a flowing wash buffer (phosphate buffer saline − 0.05% Tween 20 (PBS-T20)). The wash buffer was then replaced (under flow) with lysis buffer (1% Triton X-100 in PBS with Halt protease inhibitor mix), a process further promoted by MB AC field activation, for 30 min.

AC field-promoted exosome extraction efficiencies were compared to those achievable in the same microfluidic configuration in the absence of AC magnetic field at similar flow rates and incubation times (30 incubations at a flow rate of 2 μL/min). NTA analyses (see Materials and Methods, SI) indicated that the optimized AC magnetic field consistently supported >70% isolation efficiency of either L1CAM+ or CD9+ exosomes from diluted pooled human serum (diluted to bring exosomes within the working dynamic range of a NanoSight NS300) in 30 min compared to a <20% capture efficiency in the absence of field at 30 min incubation (Figure 1D).

### Electrochemical Analysis

**Figure 2:**
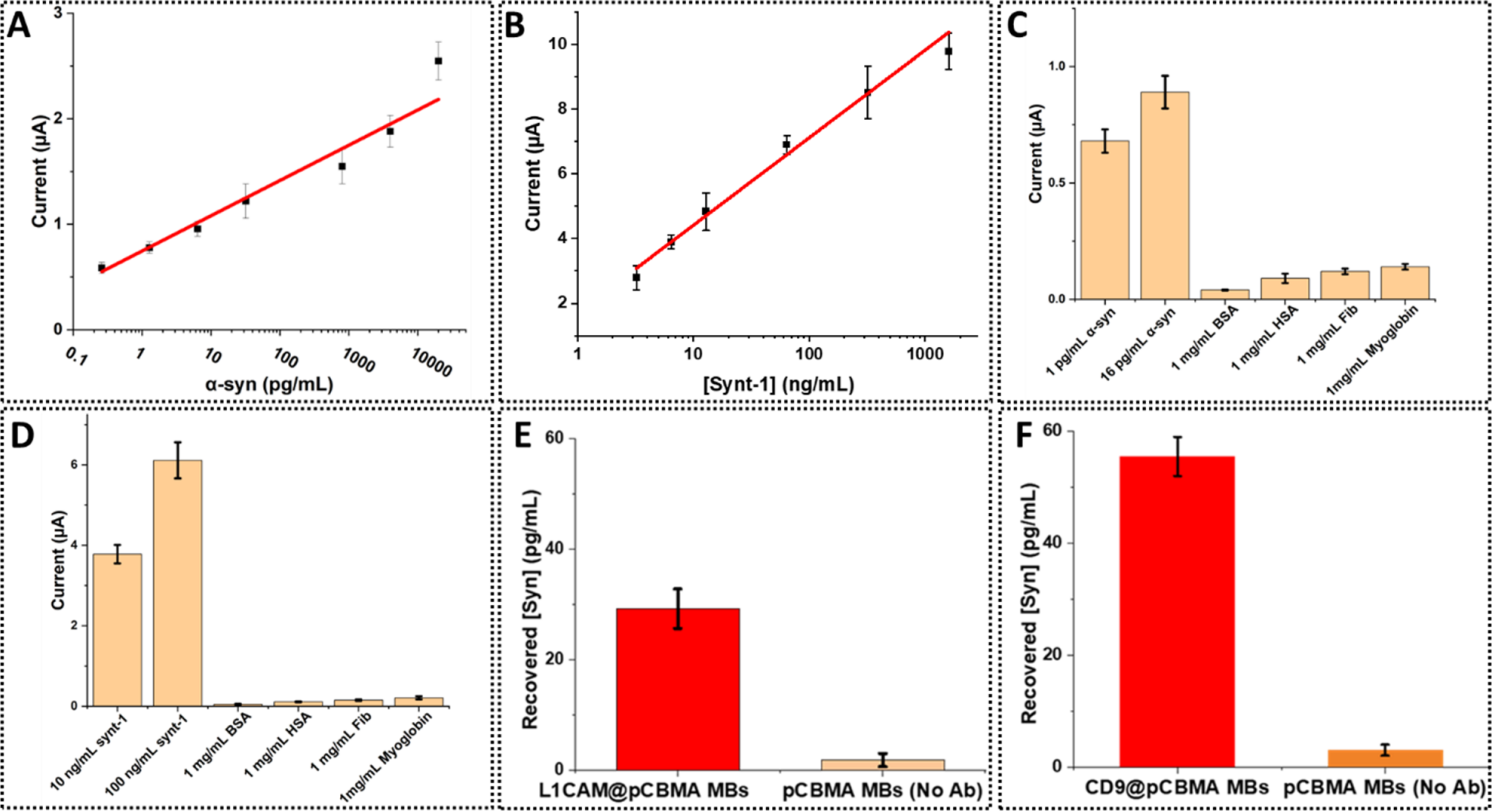
Calibration data for (A) α-syn and (B) synt-1 aliquoted in 1% BSA. Peak currents are calculated from the height of SWV signals measured 0.0–0.5 V vs Ag/AgCl screen-printed reference electrode and carbon counter electrode. (C) Specificity study of anti-α-syn-coated electrodes when challenged against common interfering proteins, and (D) same for anti-synt-1 modified electrodes. Lysates of exosomes extracted from 50 μL of pooled human serum on (E) L1CAM-functionalized pCBMA-coated MBs and (F) CD9-functionalized pCBMA-coated MBs were analyzed for α-syn content and results compared to those obtained from lysate extraction with bare pCBMA-coated MBs. Both L1CAM and CD9@pCBMA MBs showed α-syn levels consistent with expectations,^40^ while recruitment at bare pCBMA MBs was negligible (∼background signal). Error bars represent standard deviation across four independent measurements.

A duplex assay for α-syn and synt-1 (assaying both antigens simultaneously from the same sample) employing a standard sandwich electrochemical ELISA format was established and optimized.^43^ These involved the sequential incubation of the sample (15 min), biotinylated detection antibody (Ab_2_) (10 min), streptavidin-horseradish peroxidase (St-HRP) (10 min), and precipitating TMB (2 min). The HRP catalysis generates a localized concentration-dependent precipitation of oxidized TMB on the electrode surface that can be quantified from SWV peak currents.^44^ The used screen-printed 32-electrode arrays were integrated within bottomless 32 ELISA well plates (Figure S4,SI) with quantification standard deviations of <20% interarray SD and <12% interelectrodes (within the same array) for both α-syn and synt-1 (Figure S5, SI). Each well represents a separate electrochemical cell containing reference, counter, and working electrodes that are connected individually to a multipotentiostat for electrochemical measurements. Each plate was divided into two parts: 16 wells for α-syn and 16 for synt-1, enabling the analysis of both antigens across potentially eight samples. Prior to real sample analysis, arrays were calibrated using standard recombinant α-syn and synt-1 (in 1% BSA as a surrogate for the protein-rich matrix). Assays required 50 μL of sample/well and <40 min in supporting detection limits of 200 fg/mL for α-syn and 3.2 ng/mL for synt-1 and dynamic ranges spanning 0.25–5000 pg/mL for α-syn and 16–1600 ng/mL for synt-1 (Figure 2A,B). Assay selectivity was excellent when challenged against high (clinically relevant) concentrations of common interfering proteins (BSA; human serum albumin (HSA); fibrinogen; and myoglobin) (Figure 2C,D).

Once established, this assaying platform was initially applied to the analysis of exosomal α-syn and synt-1 from pooled human serum samples to further evaluate the selectivity of MB isolation. A specific focus here was a demonstrated ability to selectively isolate α-syn (of neuronal exosome origin) without interference from free α-syn in serum samples.^45^ Nonimmune-modified magnetic beads recruited very low levels of α-syn (very close to the assay background noise below its detection limit) in marked contrast to isolation with antibody modified MBs (Figure 2E,F). These findings support the previously noted NTA analyses where bare MBs showed no tendency to absorb exosomes (Figure 1A,B), while confirming the absence of any significant contribution of free α-syn within electrochemical assays.

### Assay Validation

**Figure 3:**
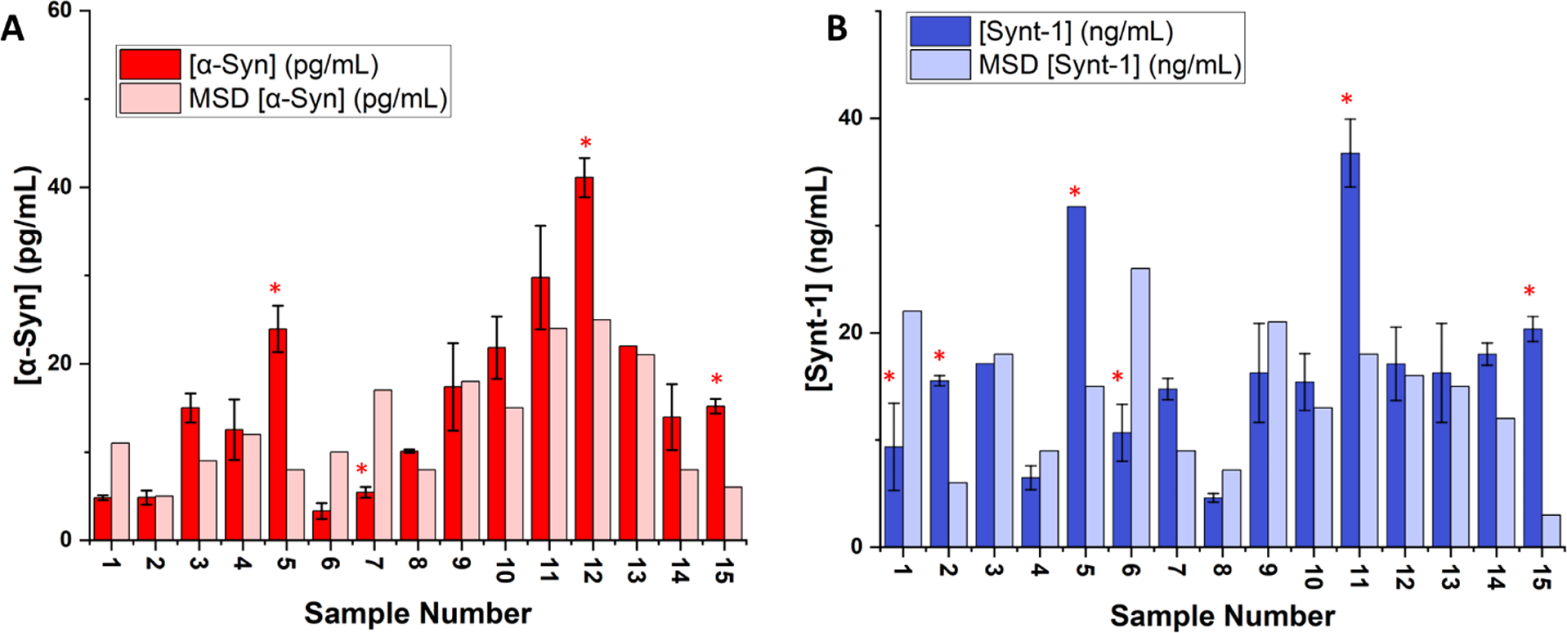
Comparative analysis of 15 control patient serum samples for (A) α-syn and (B) synt-1 by electrochemical arrays and MSD after extraction from L1CAM+ neuronal exosomes. Comparison of the results from electrochemical analysis and MSD using Student’s *t*-test showed no significant difference for α-syn (*t* = 0.93) and synt-1 (*t* = 0.98). Error bars represent standard derivation across two independent measurements. * represents samples with significant difference between the two techniques (*P* < 0.05).

To validate the performance of these analyses, 15 control samples were assayed for α-syn and synt-1, with electrochemical array quantifications (after AC microfluidic extraction) compared to those obtained from a conventional extraction protocol (overnight incubation under continuous stirring) and MSD analysis (see Materials and Methods, SI). A pleasingly good analytical correlation (considering the extraction and analytical differences) was observed with intercepts near the origin and slopes near unity (slopes 0.86 (*R*^2^ = 0.78) and 0.91 (*R*^2^ = 0.68) for α-syn and synt-1, respectively, with associated intercepts of 0.06 (±0.02) and 0.02 (± 0.03)). An individual sample comparison indicated that 75% had no significant difference (*P* < 0.05) when analyzed for α-syn and 60% had no significant difference (*P* < 0.05) in synt-1 expression levels (Figure 3A,B). One would, of course, fully expect a degree of variation with real patient sample analysis by entirely independent assaying methods (and also extraction parameters).^46,47^

### Patient Sample Analysis

The on-chip AC-assisted capture and downstream electrochemical analysis was then applied to a further 72 samples from two disease groups that we had previously found to exhibit increased α-syn levels in neuronal exosomes using MSD:^12^ PD patients (*n* = 20) versus their corresponding controls (*n* = 20) or individuals with REM sleep behavior disorder (RBD, *n* = 23) that are at risk of developing PD versus controls (*n* = 9).

**Figure 4:**
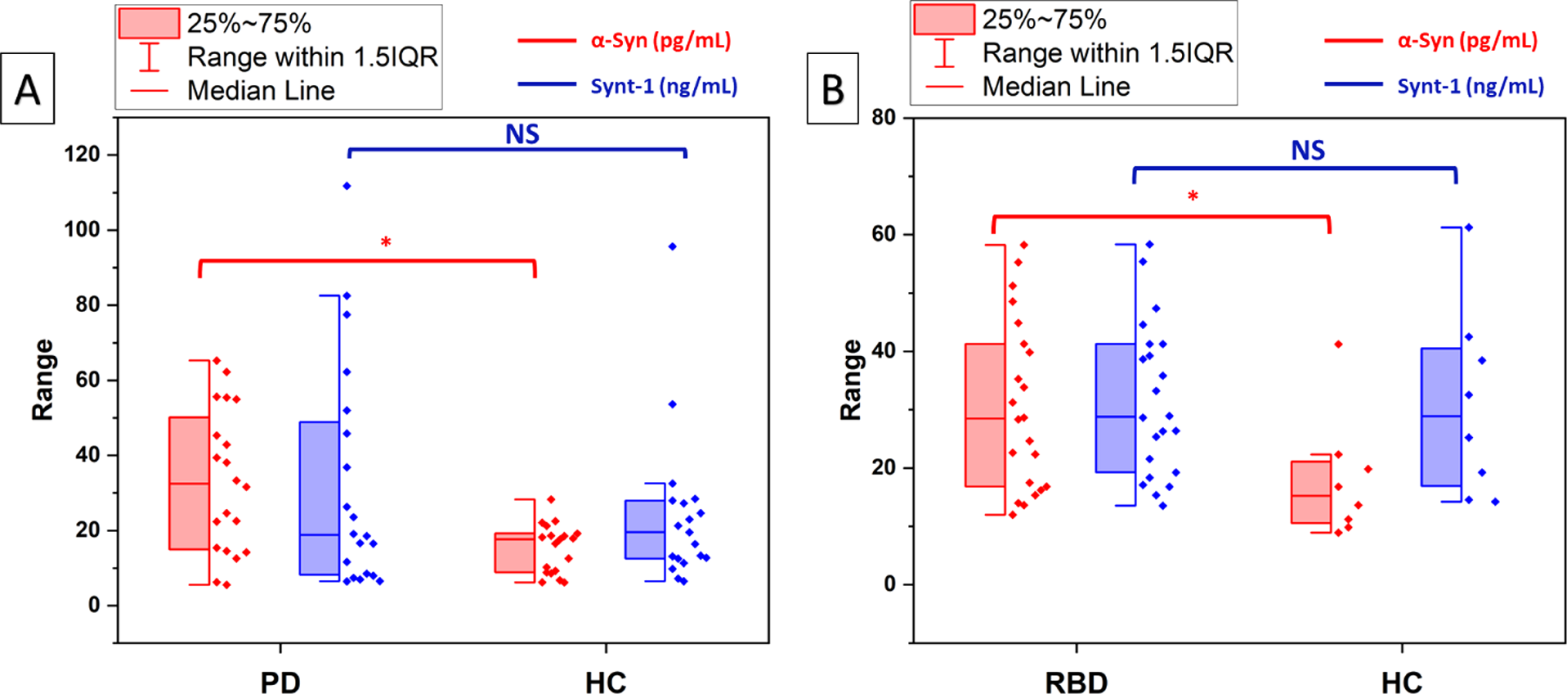
Box plot analysis of (A) 20 PD samples against their corresponding controls (*n* = 20) and (B) RBD samples (*n* = 23) and controls (*n* = 9, with one outlier) as assayed electrochemically after AC extraction for α-syn (black) and synt-1 (red). The analyses resolved a significant difference in the exosomal α-syn concentration across both cohorts. * indicates a significant difference between the two means when evaluated by a *t*-test (*P* < 0.05). NS = no significant difference.

For both groups, the AC exosome isolation with downstream multiplexed electrochemical assaying showed a statistically significant difference in the resolved expression of α-syn in PD or RBD patients compared to age-matched controls as depicted by the box plot analysis of the recovered concentrations (Figure 4A,B). There was no significant difference in the expression of synt-1 for either groups consistent with our previous finding^40^ (Figure 4A,B). The box plot analysis also supported the exclusion of outliers (points outside 1.5 × interquartile range (IQR)); there were specifically three outliers within the synt-1 data, as presented in Figure 4A, and two outliers within the HC group, as shown in Figure 4B.^48^ The higher distribution of synt-1, which was observed in the PD group when compared to the RBD group, may be due to differences in the sample collection procedure or the racial/ethnic differences between the two cohorts.

**Figure 5:**
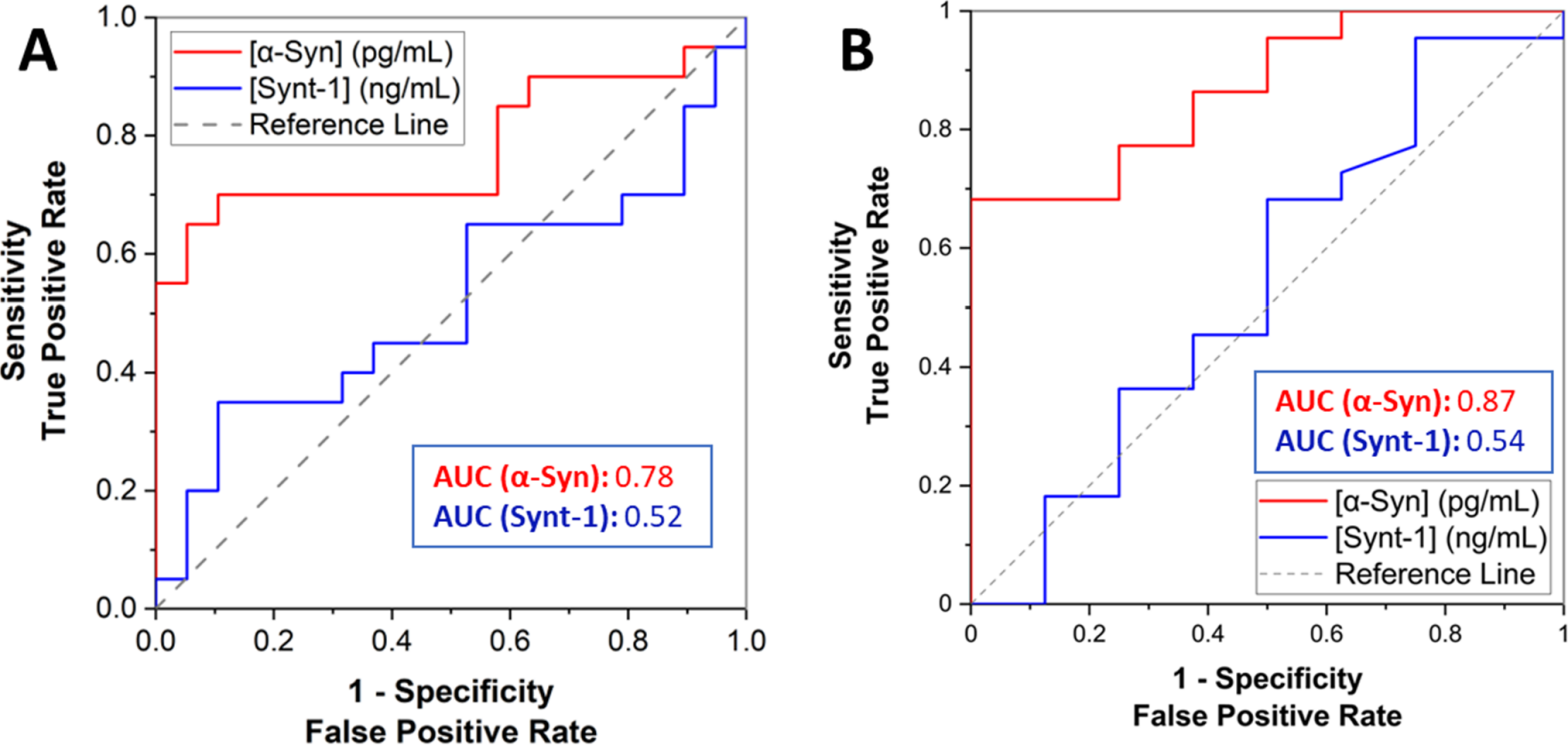
ROC plots and corresponding AUC values from α-syn and synt-1 analyses after AC-assisted extraction with electrochemical analysis of (A) 20 PD samples and their corresponding 20 HC from the PMMI cohort, (B) 23 RBD samples and their corresponding 9 HC. The dashed reference line indicates neutral operators and an AUC of 0.5.

In order to assess the clinical validity of the proposed AC field extraction/electrochemical analysis in detecting PD, the receiver operating characteristic (ROC) of the results obtained for both RBD and PD cohorts were analyzed. The area under the curve (AUC) value was resolved to be 0.78 for the PD cohort-assayed α-syn (Figure 5A). The AUC of the same analysis was >0.85 for the α-syn for samples obtained from the RBD cohort (Figure 5B). Synt-1 data analyses (Figure 5A–D) revealed a low AUC, which is indicative of its expected low clinical value as this is a generic marker of exosomes. These findings are comparable to our previously reported AUC = 0.86 using overnight immunocapture and downstream standard MSD analysis of α-syn in *n* = 664 serum samples from independent clinical cohorts.^12^

The results herein demonstrate how the application of an alternating magnetic field can improve the immunomagnetic extraction of target neuronal exosomes from serum samples prior to the ultrasensitive and clinically valuable electrochemical array analysis of the relevant cargo marker. Applied here to α-syn, the configuration was able to support a robust isolation and quantification from <50 μL of sample in less than 75 min total time. The use of zwitterionic polymer-coated MBs ensured that downstream assays were possible without interference from free serum proteins. Significantly, the MBs showed no tendency to nonspecifically adsorb either free α-syn when exposed to clinically relevant concentrations (Figure 2E,F) or exosomes from serum samples (Figure 1D), making false-positive analyses unlikely.^45^

In line exosome lysis releases the protein cargo that can be assayed using a highly sensitive multiplexed electrochemical ELISA on 32 screen-printed electrodes housed within standard bottomless ELISA microwell plates. Applied here to the blind analysis of 72 samples from two different patient cohorts, results correlate very well with those achieved from the same patient samples by more standard MSD (multiday, large volume) analyses. They also validate, through a totally independent methodology, our previous finding of neuronal exosome α-syn as a biomarker for the prediction or diagnosis of PD.^12^ Mean recovered concentrations of neuronal α-syn from the PD cohort was approximately 200% that of the recovered α-syn concentrations from HC patients, supporting its value in PD identification, a finding further confirmed by an AUC > 0.75 from ROC analyses.^13^

## Conclusions

An on-chip magnetically promoted extraction of neuronal exosomes using anti-L1CAM antibody-coated MBs from serum samples coupled to downstream electrochemical analysis of α-syn and synt-1 was developed. The configuration encompasses a continuous dynamic capture particle-serum mixing powered by a tuneable AC magnetic field controlling the bidirectional movement of nonfouling MBs. Highly selective exosome extraction from serum is promoted with negligible contribution from the background within minutes from low sample volumes. Subsequent in line lysis of magnetically trapped exosomes is then followed by sub pg/mL LOD assays. All patient analyses were rigorously cross-referenced to standard extraction and quantification methods. We believe the outlined, and generic, methodology is of value in accelerating the efficiency and throughput of scaleable exosome diagnostics.

## Supporting Information Available

Materials and methods, microfluidic chip design, MB characterization, tyramide signal amplification, electrochemical ELISA plate design, reproducibility studies, and MSD analysis (PDF)
