# Supplementary material for: Alternating Magnetic Field-Promoted Nanoparticle Mixing: The On-Chip Immunocapture of Serum Neuronal Exosomes for Parkinson’s Disease Diagnostics
Source: Anal Chem. 2023 May 11;95(20):7906–13. doi: 10.1021/acs.analchem.3c00357 (PMC10209966; doi:10.1021/acs.analchem.3c00357)
Supplement: Supplementary file 1 — ac3c00357_si_001.pdf [file ac3c00357_si_001.pdf]

Supporting Information

Alternating magnetic field-promoted nanoparticle mixing: the on-chip immunocapture of serum neuronal exosomes for Parkinson's disease diagnostics

Mohamed Sharafeldin,[a,b] Shijun Yan,[c,d] Cheng Jiang,[a,c,d] George K Tofaris[c,d] and Jason J. Davis [a*]

[a]*Department of Chemistry, University of Oxford, South Parks Road, Oxford OX1 3QZ, UK*
[b]*Department of Chemistry, University of Otago, Dunedin 9054, New Zealand*
[c]*Nuffield Department of Clinical Neurosciences, John Radcliffe Hospital, University of Oxford, Oxford OX3 9DU , UK*
[d]*Kavli Institute for Nanoscience Discovery, Dorothy Crowfoot Hodgkin Building, University of Oxford, Oxford OX1 3QU, UK*
** Corresponding Author*

Table of Contents

Materials and Methods 2
- Materials and equipment 2
- Calculation of antibody coverage 2
- Nanoparticle Tracking analysis (NTA) 2
- Zeta potential and dynamic light scattering 2
- Calculation of Isolation efficiency 2
- Immunofluorescence Assays 3
- Patient population. 3
- Electrochemiluminescence analysis 3

Design of the 3D printed microfluidic chip 4
Characterization of the pCBMA coated magnetic beads 5
Tyramide signal amplification (TSA) assays 6
Electrochemical ELISA plate design 7
Reproducibility study for α-syn and synt-1 electrochemical assay 8
Unprocessed Western blot 9

Materials and Methods

Materials and equipment. Poly(carboxybetaine methacrylate) (pCBMA)-magnetic beads were synthesized as reported previously.[1] Anti-L1CAM antibody (Abcam, ab20148), anti-CD9 antibody (Insight Biotechnology, TA320303), and anti-IgG antibody Dylight 680 were obtained , respectively. Bovine Serum Albumin (BSA), N-Hydroxysuccinimide (NHS), and N-Ethyl-N′-(3-dimethylaminopropyl)-carbodiimide hydrochloride (EDC) were purchased from Sigma-Aldrich. Phosphate buffer saline (PBS, pH=7.4) was prepared freshly and filtered through 0.22 μm filters. Serum samples were pooled from 20 different patient's samples without further purification or pre-treatment. Nanoparticle tracking analysis (NTA) was performed using Malvern NanoSight NS500 (Malvern, UK), configured with a 405 nm laser and a high sensitivity CMOS camera (OrcaFlash2.8, Hamamatsu C11440, NanoSight Ltd.). Videos were collected and analysed using the NTA software (version 3.2, build 0025) with detection threshold set at 5. All analysis was carried out at a room temperature. All comparative studies for calculation of capture efficiency were performed using same parameters (e.g., camera level and Focus). By monitoring the trajectory of movement, particle number and size distribution within the range of 40-140 nm were estimated. For consistent reading, the measurement settings were optimized and three replicas/sample were performed to obtain the averaged measurements.

Calculation of antibody coverage. The antibody coverage was calculated at 4.8 μg CD9 antibody per 1 mg of beads and 2.36 μg L1CAM antibody per 1 mg of beads (equal to 7.0×10^4 CD9 antibodies on 1 bead which occupy 80% of antibody surface and 1.9×10^4 L1CAM antibodies on 1 bead which occupy 30% of antibody surface, both compared to theoretical value for 1 μM bead) using a BCA assay. In this manner, two batches of MBs were prepared in similar condition, where one batch was modified with Ab and the other was bare MBs (blank). Total protein analysis of both batches was performed by incubating samples with bicinchoninic acid (BCA) at 37 C for 30 min and measuring the resulting absorbance at 562 nm. The BCA assay was also performed using standard antibodies diluted in PBS and the absorbance of the Ab-coated MBs was used to estimate the number of MB bound antibodies.

Nanoparticle Tracking analysis (NTA). All samples were diluted 500x in particle free PBS to a final volume of 1 ml to bring the concentration of the exosomes into the operating dynamic range of NS300. Imaging parameters were adjusted according to the manufacturer's recommendation (NanoSight NS300 User Manual, MAN0541-01-EN-00, 2017). 1 mL sample was added int the microfluidic chip and three 1-min videos were captured at 25°C and analysed by the in-build NanoSight Software NTA version 3.2, build 0025 with a detection threshold of 5. Each sample was run in three replicates (3 mL total volume) and the number of exosomes estimated from the average across the 9 different measurements.

Zeta potential and dynamic light scattering. The surface potential and hydrodynamic diameter of MBs (at 1 mg/mL in PBS buffer pH 7.4) was measured using Malvern© Zetasizer nano with a 532 nm laser.

Calculation of Isolation efficiency. The MBs were collected after extracting exosomes and washed 3X with PBS. MBs were then incubated for 10 min with glycine-HCl buffer (pH=2.00),

then neutralized with Tris buffer at pH= 8.00. The MBs were separated on application of a permanent magnet and the supernatant collected for NTA. Finally, the capture efficiency was calculated by dividing the number of exosomes released from MBs/total number of exosomes in the diluted serum) ×100% (within 40-140 nm vesicle diameter).

Immunofluorescence Assays. Immunomagnetic beads loaded with neuronal EVs are collected from the microfluidic extraction chips and washed 3 times with buffer. The presence of rabbit L1CAM antibodies or CD9 antibodies (that reflect the number of exosomes adsorbed on the MBs) was evaluated using anti-rabbit Alexa Fluor™ 488 SuperBoost™ tyramide signal amplification (TSA) Kit (B40922, Thermo Fisher Scientific): a sensitive assay for rabbit IGg antibodies down to picomolar concentrations. MBs were allowed to fix on a glass slide using 3% glutaraldehyde solution in water. Fixed beads were then incubated with the rabbit CD9 antibody or rabbit L1CAM antibodies (10 µg/mL in 0.1% BSA in PBS buffer) for 2h. Glass slides were washed with PBS-T20 and incubated for 1 hr with HRP-anti-rabbit IGg antibodies, washed and incubated with tyramide-Alexa Fluor 488 complex, washed, and imaged using fluorescent microscope (ZEISS©). Same procedures were perfumed using unfunctionalized (bare) pCBMA MBs incubated with exosomes (extracted from cell culture media). The bare pCBMA MBS showed no immunoreactivity to the TSA anti-rabbit kit in contrast to L1CAM coated MBs fixes on glass slides with similar assay (Figure S3, SI)

Patient population. Serum samples from a total of 72 subjects were analysed from two cohorts: (1) n=23 individuals with polysomnographically confirmed rapid eye movement sleep behaviour disorder (RBD) and n=9 controls from the Oxford Discovery Cohort or the Oxford Radcliffe Biobank and (2) n=20 PD and their corresponding controls (n=20) from the Parkinson's Progression Markers Initiative (PPMI). The Oxford Discovery cohort was approved by the South-Central Oxford A Research Ethics Committee (IRAS 188167) and the Oxford Radcliffe Biobank as approved by the South-Central Oxford C Research Committee (19/SC/0173). Written informed consent was obtained from all participants or their caregivers.

Electrochemiluminescence analysis. The Electrochemiluminescence (ECL) was performed similarly to a method we previously described.[2] ECL was performed in 96-well Meso Scale Discovery (MSD) U-Plex plates that enable multiplexing of markers in the same exosome preparation. All steps were performed at room temperature. Two unique linkers for the selected markers (syntenin-1 and α-synuclein) were used according to the manufacturer's protocol. After three washes, detection antibodies with Sulfo-TAG-labelling were incubated for 1 hour. Following washes with PBS-T20 and addition of MSD Read buffer the plates were read using the MSD-ECL platform (QuickPlex SQ 120) and data were analysed with the MSD Discovery Workbench 3.0 Data Analysis Toolbox. Antibody pairs for α-synuclein were provided by MSD and preconjugated with biotin and ruthenium tag. Additive-free anti-syntenin-1 goat polyclonal antibody (PAB7132, Abnova) and anti-syntenin-1 rabbit monoclonal antibody (ab236071, Abcam) were conjugated with biotin and ruthenium and used as capture and detection antibodies, respectively. For combined exosomal α-synuclein and syntenin-1, we used a duplex MSD that we previously demonstrated to specifically detect these markers in immunocaptured exosomes. [2]

Design of the 3D printed microfluidic chip

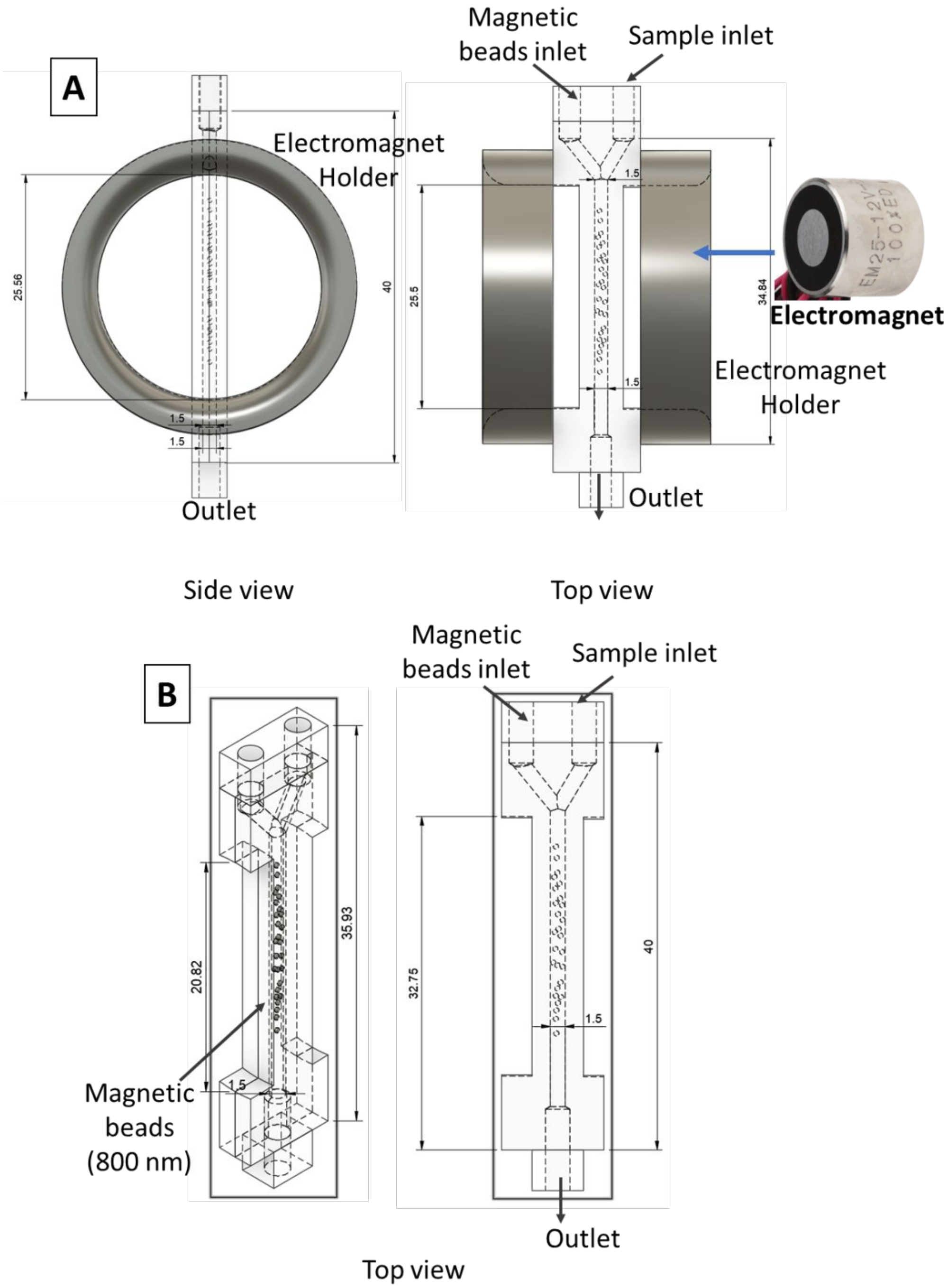

Figure S 1: detailed design of the 3D printed microfluidic mixer. (A) side and top view of the chip with the 25 mm diameter electromagnet housing. Two ring housings are designed to precisely contain two electromagnets on both sides of the flow channel (B). The microfluidic flow channel (1.5 mm diameter and 35 mm length) is equipped with two inlets for the introduction of sample and magnetic beads and an outlet.

Characterization of the pCBMA coated magnetic beads

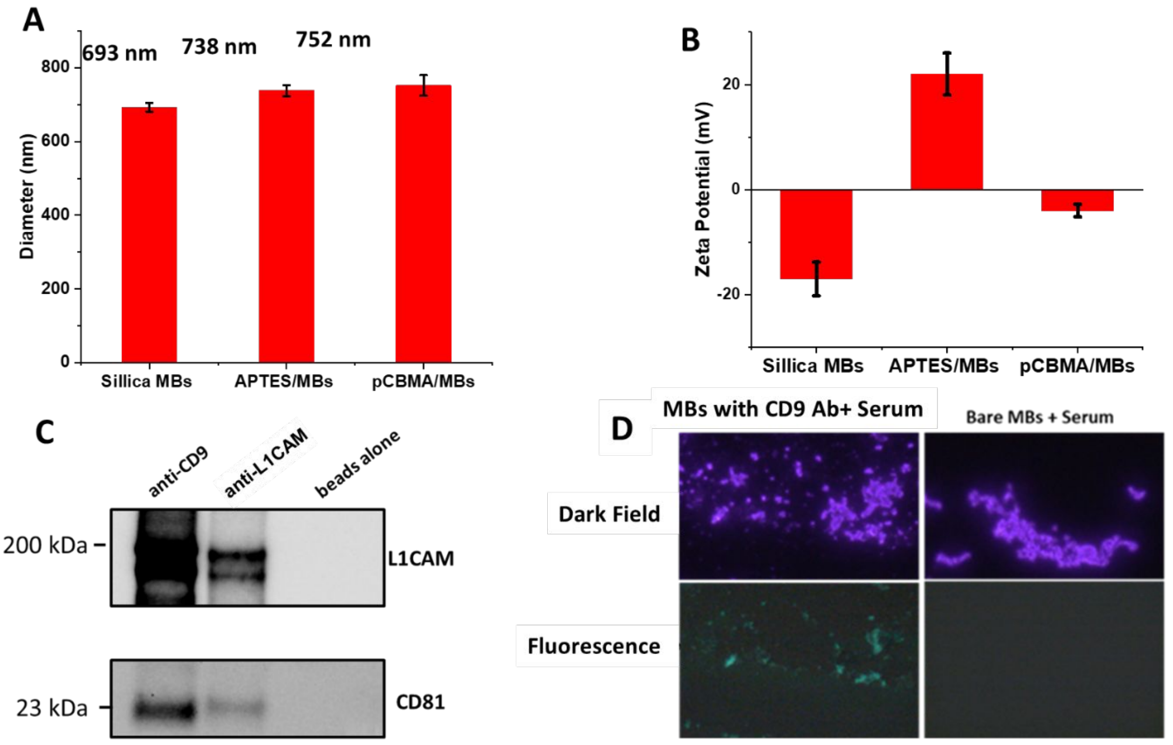

Figure S 2: Characterization of the size (A) and surface zeta potential (B) of the MBs after each surface modification step. Error bars represent standard deviation across three independent measurements (C) Western blot analysis of exosome specific proteins from lysed extracts of exosomes isolated from 250 µL of serum samples using Ab-modified beads against bare magnetic beads; detectable protein is only present in antibody modified beads while proteins on bare MBs were below WB detection limit; indicating excellent anti-fouling properties (see Figure S6 for raw unprocessed western blots). (D) Fluorescence microscopy analysis of non-specific adsorption of exosomes onto bare MBs compared to anti-CD9 coated MBS. After incubating MBs (bare or antibody-modified) with 50 µL fractionated exosome samples from tissue culture for 2 hrs, the MBs were washed and fixed on the glass slides. Beads were then incubated with rabbit anti-CD9 antibodies, washed and incubated with anti-rabbit IGg labelled with HRP. Washed slides were then incubated with tyramide-Alxa Fluor 488 in presence of hydrogen peroxide. Ab-modified magnetic beads were able to capture exosomes form samples, as indicated by the fluorescence signal, while bare MBs showed no fluorescence indicating an absence of adsorbed exosomes.

Tyramide signal amplification (TSA) assays

This assay was employed to test the presence of any non-specifically adsorbed exosomes on bare MBs. The test encompasses the use of HRP-assisted amplified fluorescence using the tyramide-Alexa Fluor 488 complex. Anti-rabbit IGg labeled with HRP are allowed to incubate with fixed MBs that were previously incubated with exosomes isolated from tissue culture media using size exclusion chromatography (SEC). The presence of exosomes on MBs will elicit the capture of exosome-specific antibodies (rabbit anti-CD9) that capture the HRP-anti-rabbit-IGg. The HRP catalyzes deposition of tyramide on and near a target protein in presence of low concentration of hydrogen peroxide. This test is extremely sensitive, facilitating target molecules down to few attomoles.

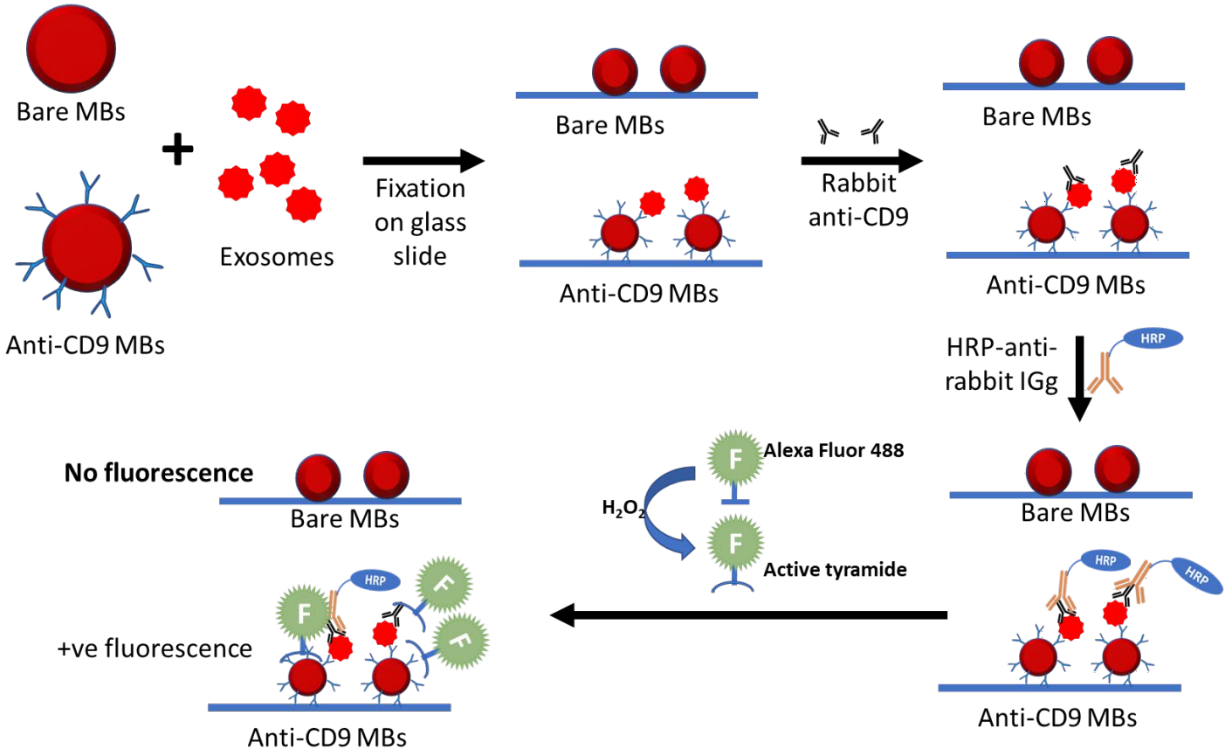

Figure S 3: Schematic depiction of the tyramide signal amplification (TSA) fluorescence assay for the detection of non-specifically adsorbed exosomes on bare MBs as compared to anti-CD9 modified MBs.

Electrochemical ELISA plate design

All electrochemical measurements were performed on a 32-electrodes array housed in standard bottomless ELISA plate. Each well is designed to house an independent electrochemical cell with a 3.0 mm diameter working screen-printed carbon electrode, Ag/AgCl counter electrode and screen-printed carbon counter electrode.

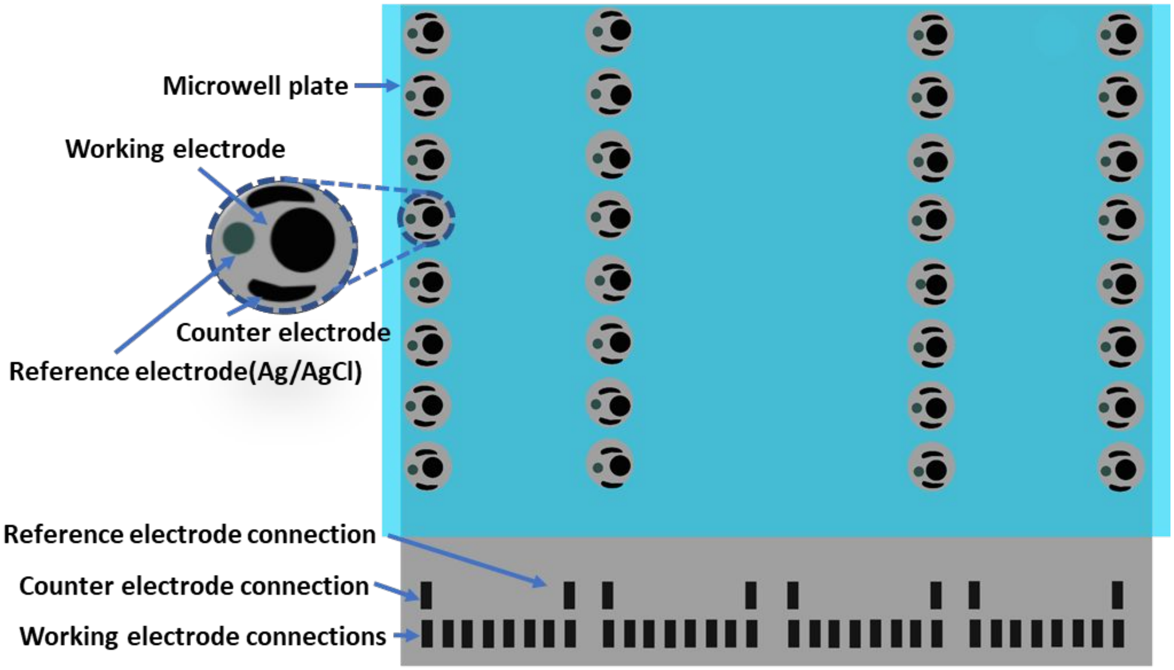

Figure S 4: Schematic depiction of electrode array. The array is designed to fit into a bottomless standard 96 micro well-plate in a way such that each well houses a 3 mm screen-printed carbon electrode, a screen-printed carbon counter electrode and Ag/AgCl reference electrode.

Reproducibility study for α-syn and synt-1 electrochemical assay

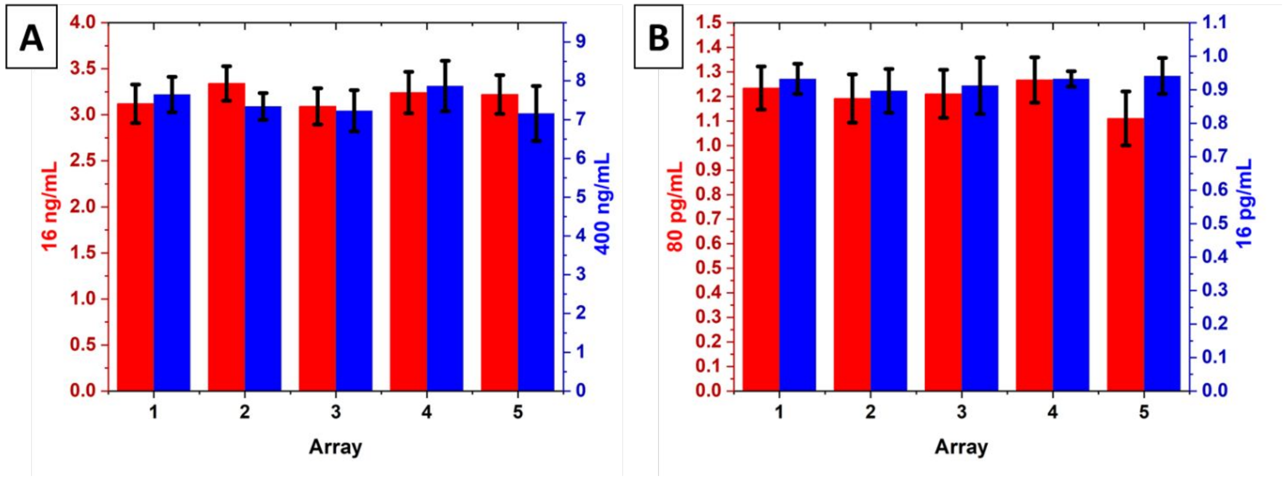

Figure S 5: Reproducibility analyses generated by running two concentrations of (A) synt-1 (16 ng/mL and 400 ng/mL) and (B) α-Syn (128 fg/mL and 16 pg/mL) for 5 days over different electrode arrays. Each measurement represents an average across 6 individual electrodes (n=6). Error bars represent standard deviation across 6 different measurements.

Unprocessed Western blot

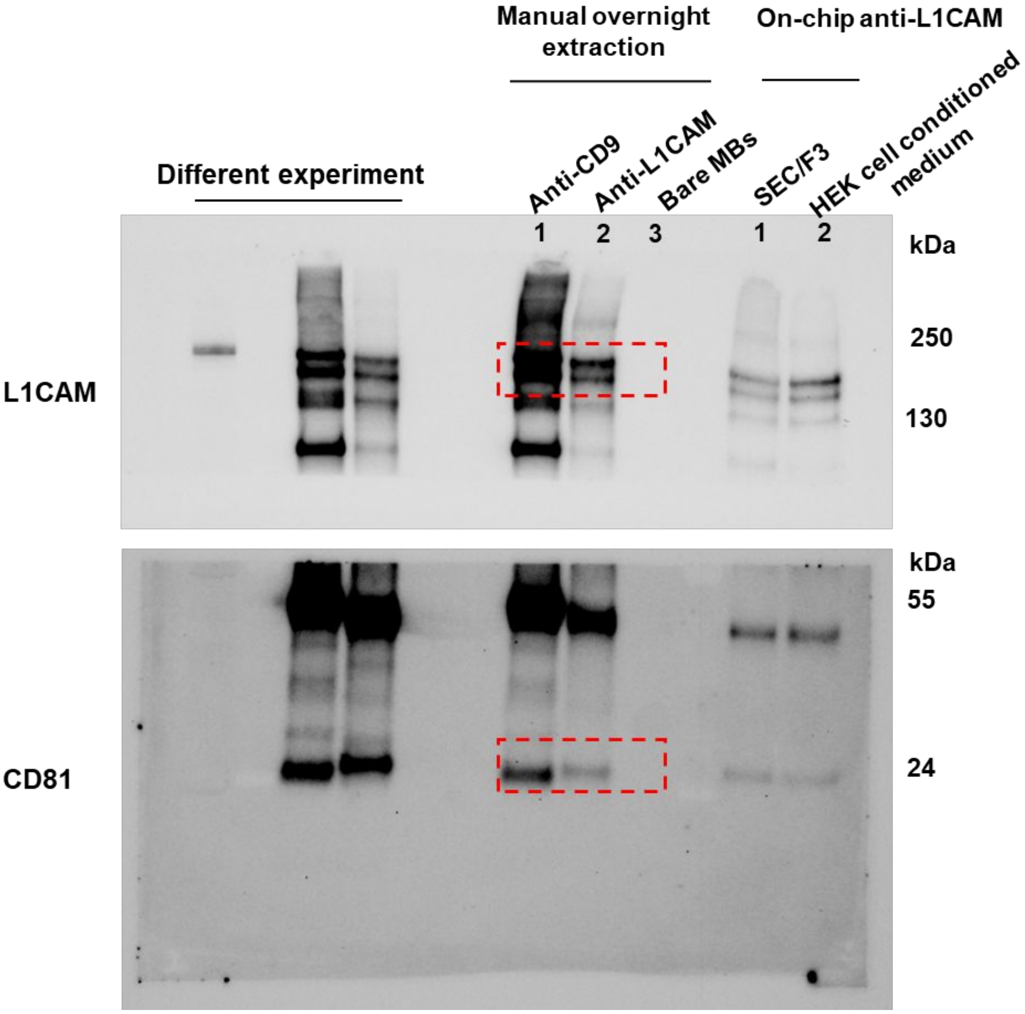

Figure S 6: Raw, uncropped, and unprocessed western blots showing area of interest highlighted in red squares (presented in Figure S2). The first 3 columns correspond to an unrelated experiment (not within the scope of this article). The middle 3 columns (numbered 1-3) represent bands from lysates of exosomes isolated from 250 µL of whole serum samples using Ab-modified beads against bare magnetic beads after overnight incubation under stirring. The last 2 columns represent lysates of exosomes extracted using on-chip AC magnetic field assisted microfluidic isolation from either fractioned serum (using size exclusion chromatography, SEC) or HEK cell conditioned medium using anti-L1CAM coated MBs.

References

(1) Fu, Y.; Jiang, C.; Tofaris, G. K.; Davis, J. J. Facile impedimetric analysis of neuronal exosome markers in Parkinson's disease diagnostics. *Anal. Chem.* **2020**, *92* (20), 13647.
(2) Jiang, C.; Hopfner, F.; Katsikoudi, A.; Hein, R.; Catli, C.; Evetts, S.; Huang, Y.; Wang, H.; Ryder, J. W.; Kuhlenbaeumer, G.; et al. Serum neuronal exosomes predict and differentiate Parkinson's disease from atypical parkinsonism. *J. Neurol. Neurosurg. Psychiatry* **2020**, *91* (7), 720.
